# Supplementary material for: Organic narrowband near-infrared photodetectors based on intermolecular charge-transfer absorption
Source: Nat Commun. 2017 Jun 5;8:15421. doi: 10.1038/ncomms15421 (PMC5465315; doi:10.1038/ncomms15421)
Supplement: Supplementary Information — Supplementary Figures, Supplementary Tables, Supplementary Notes, Supplementary Discussion, Supplementary Methods and Supplementary References [file ncomms15421-s1.pdf]

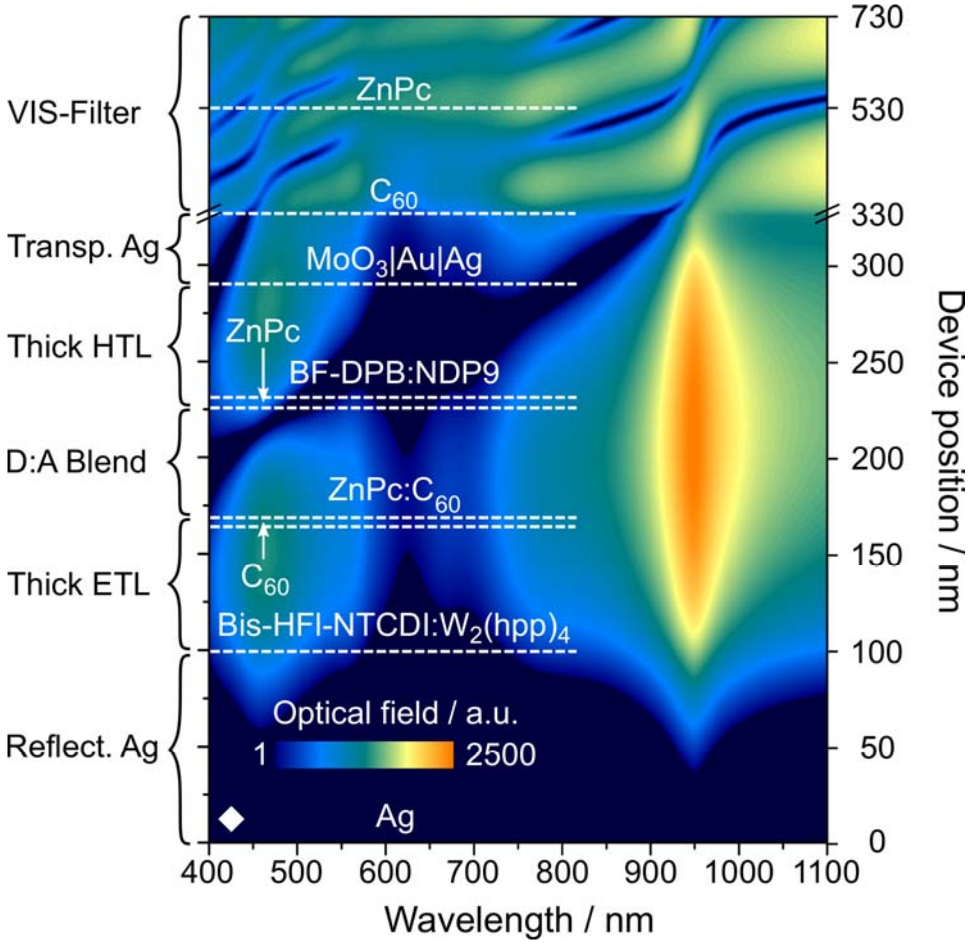

Supplementary Figure 1 | Simulated optical field distribution in a resonant cavity enhanced photodetector. The spatial and spectral dependency is shown for a ZnPc:C_{60} microcavity with a resonance wavelength of 950nm, as the one labeled with ◆ in Figure 2a and 3 in the main text. The blended absorber layer, providing CT absorption, is centered in the optical field maximum of the intended resonance wavelength. Neat layers of each blend constituent are sequentially deposited outside the microcavity to filter out the main extinction above the optical gap. The layer sequence processed onto the upper silver electrode is simplified for readability. The optical field is color-coded on logarithmic scale.

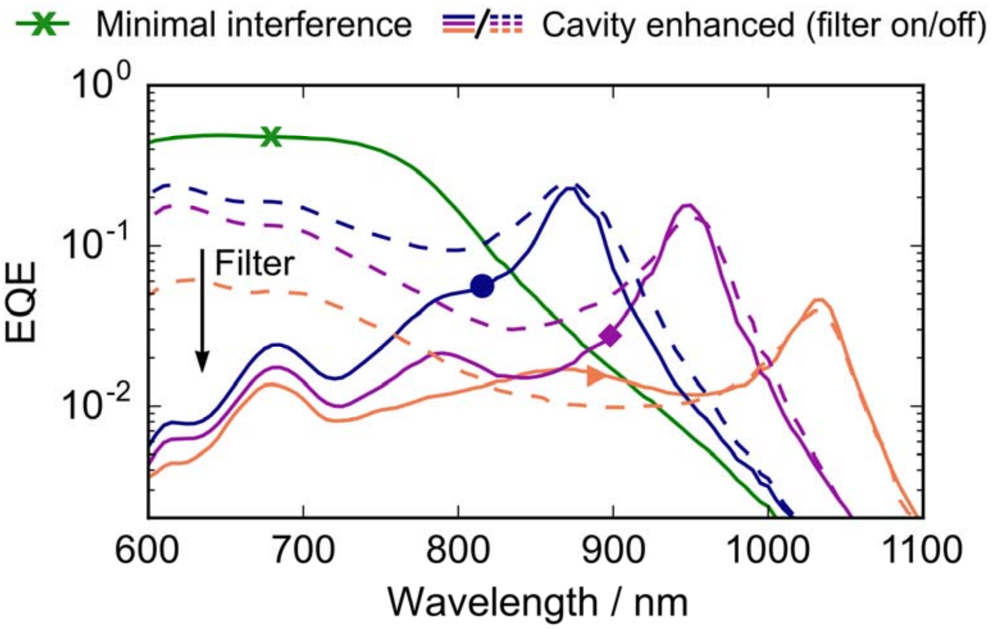

Supplementary Figure 2 | Filter effect of neat absorber sequence. The green line (X) represents the EQE of a ZnPc:C_{60} solar cell with minimized interference effect as in Figure 2 in the main text. The remaining solid lines show the EQE spectra of cavity enhanced photodetectors with resonances at 875nm (●), 950nm (◆), and 1035nm (▶), labelled as in Figure 2 in the main text. The dashed lines represent the behavior of device areas without the filter sequence. For resonances close to the absorption edge of neat ZnPc, as for 875nm (●), the cavity induced peak experiences is slightly narrowed further due to interference. This effect dampens for subgap photons such as for 1035nm (▶).

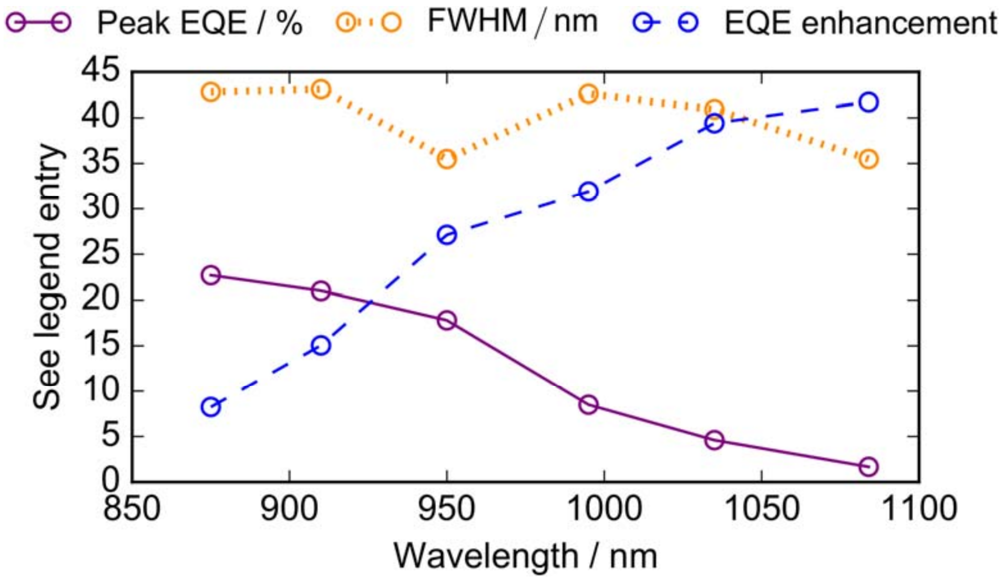

Supplementary Figure 3 | Resonance peak analysis of ZnPc:C_{60} photodetectors. In reference to Figure 2a from the main text, the respective EQE peak height, full width at half maximum (FWHM), and EQE enhancement are shown as solid, dotted, and dashed line, respectively. The latter is determined with respect to the reference device comprising thin transport layers and a highly transparent electrode for minimal interference (crossed, green line in Figure 2a in the main text or Supplementary Fig. 2). For details on the corresponding device architectures, we refer the reader to Supplementary Table 1.

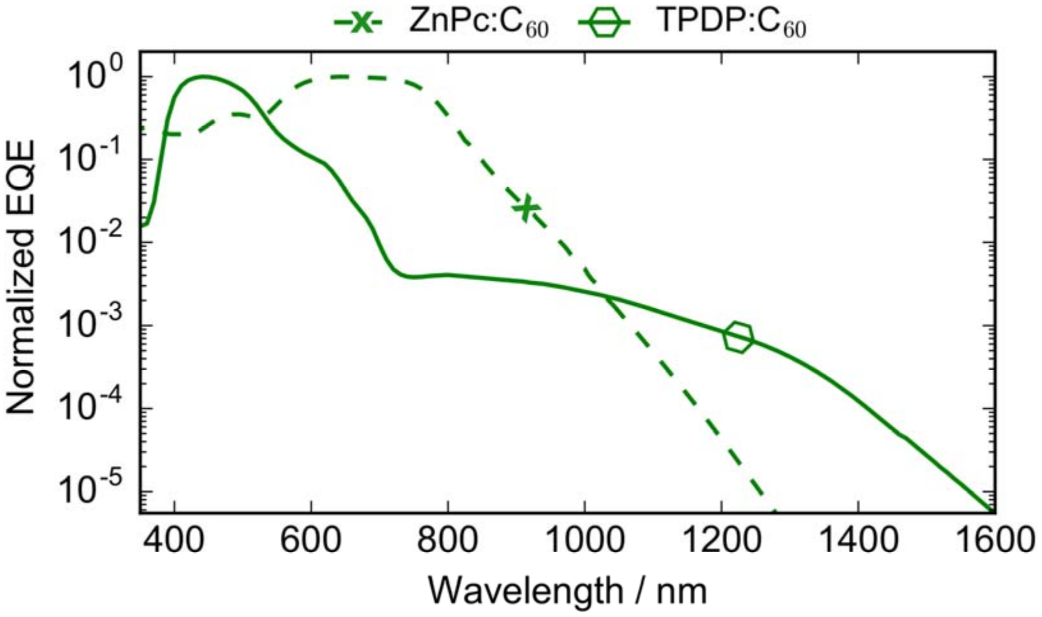

Supplementary Figure 4 | Comparison of charge-transfer absorption for ZnPc:C_{60} and TPDP:C_{60} blends. EQE spectra of devices with minimal interference effects are shown. The dashed line (X) represents a ZnPc:C_{60} blend and the solid line (⬡) a TPDP:C_{60} mixture. Both spectra are normalized for readability. The practically relevant CT absorption band for ZnPc:C_{60} extends from 850nm to 1100nm, while for TPDP:C_{60} it appears from 725nm to 1600nm. Typical extinction coefficients (imaginary part of the refractive index) observed for both materials systems range in the orders of magnitude 10^{-4} to 10^{-3}, i.e. about 100 to 1000 times lower than typical values for optical transitions in neat organic materials.

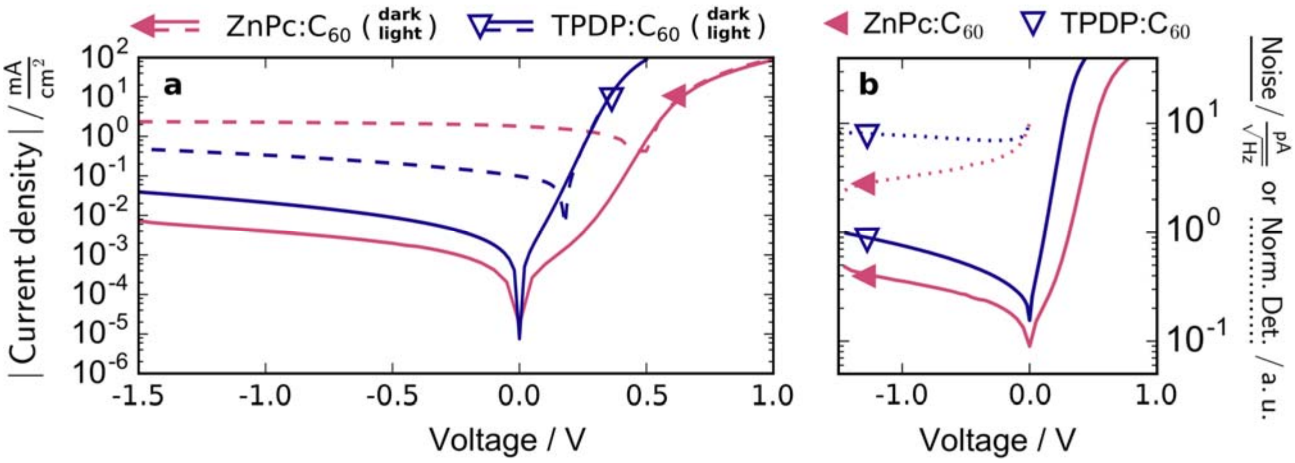

Supplementary Figure 5 | Comparison of detector voltage behavior. a: Current-voltage-characteristics. Lines with a pink ◀ (blue ▽) represent a ZnPc:C_{60} (TPDP:C_{60}) photodetector with a resonance wavelength of 995nm (1280nm), labeled as in Figure 2a (5a) in the main text. While solid lines show the behavior under dark conditions, dashed graphs are taken under excitation of a xenon lamp with 100mWcm^{-2}. According to the optimization strategies outlined in the main text, we expect room for improved dark current characteristics. **b:** Voltage dependence of the sum of shot and thermal noise as solid lines and of the normalized specific detectivity as dashed lines.

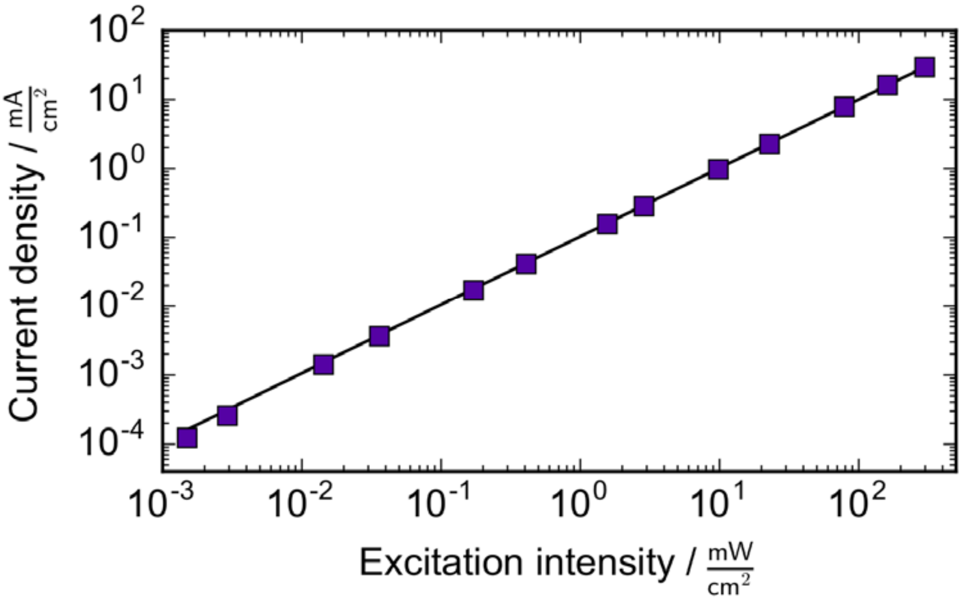

Supplementary Figure 6 | Dynamic range of a ZnPc:C_{60} photodetector. A plot of the photocurrent density versus light intensity of a ZnPc:C_{60} device with a resonance wavelength of 910nm, labeled with ■ in Figure 2a of the main text. The sample is excited at 905nm and kept at short-circuit during the measurement. A fit of the experimental data (purple ■) with a power low (black line) yields an exponent of 0.99. As the deviation of the photocurrent from a linear behavior is not yet reached for high intensities, we conclude a linear dynamic range of at least 108dB. We estimate the uncertainty for both photo-response and intensity to be 8% for each quantity. For the lowest three intensities, we obtain elevated uncertainties of 37%, 22%, and 10% (read from low to high intensity), as those measurement points are taken close the noise level of the setup.

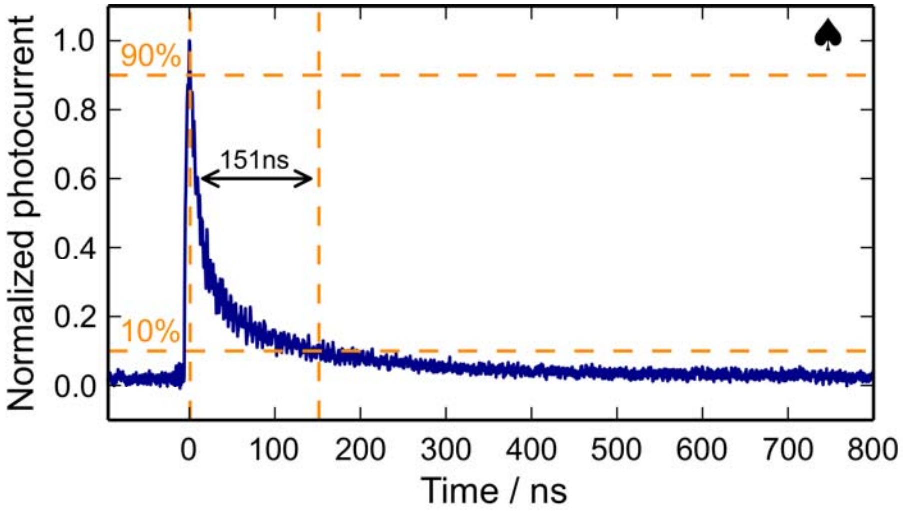

Supplementary Figure 7 | Response time of a ZnPc:C_{60} photodetector. Transient photocurrent (TPC) response of a ZnPc:C_{60} device with a resonance wavelength $\gtrsim$1100nm, labeled with ♠ as in Supplementary Table 1, excited with 25ps NIR pulses at 1064nm. The signal increases from 10% to 90% of the maximum photocurrent within 3ns and decreases from 90% back to 10% within 151ns. Hereby, the fast decay within the first 50ns is identified with the limited response dynamics of the measurement circuit featuring a resistance of 100Ω and a capacitance of 0.2nF.

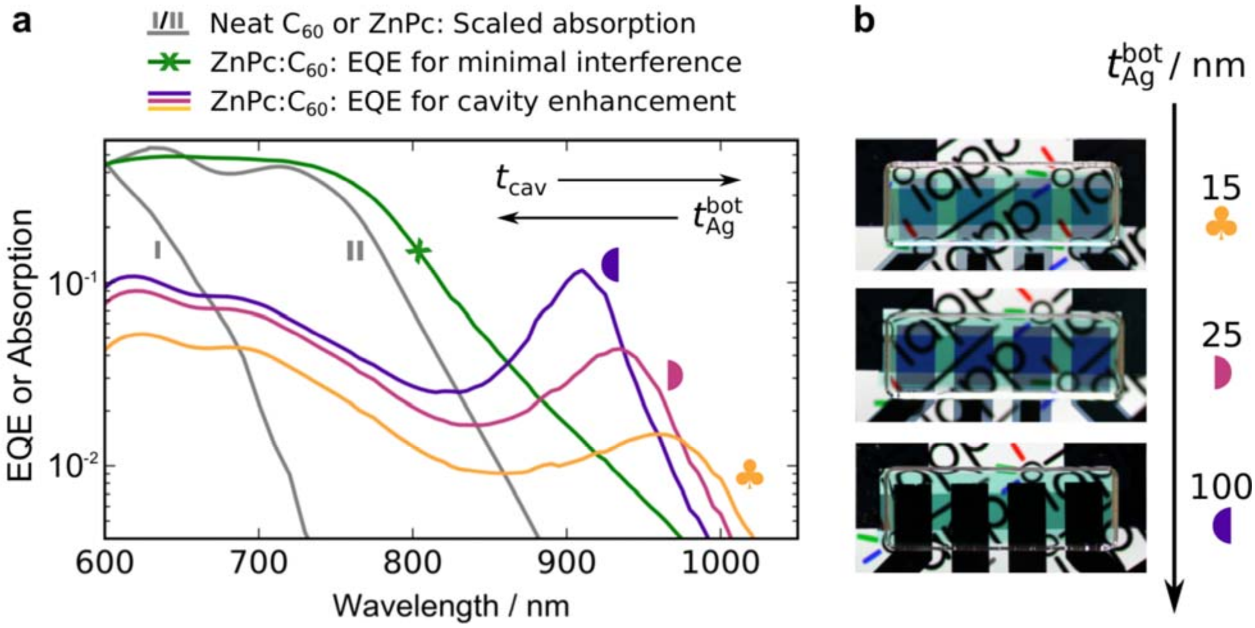

Supplementary Figure 8 | Visible transparency and alternative *p*-doping for ZnPc:C_{60} photodetectors. Further ZnPc:C_{60} photodetectors are built, whereas the hole transport layer is modified upon exchange of the proprietary *p*-dopant NDP9 by the disclosed molecule F_6-TCNNQ. Details on the corresponding layer sequences are documented in Supplementary Table 1. **a:** EQE of three photodetectors (labeled as half circles or club) with alternative *p*-doping, a ZnPc:C_{60} solar cell (X) with minimal optical cavity effect, and the scaled absorption of neat C_{60} (I) and ZnPc (II). Upon thickness variation of both transport layers, the formation as well as the spectral shift of a resonance peak are successfully reproduced, as demonstrated for resonances between 910nm and 960nm. In comparison to Figure 2a in the main text, no optical filters for the visible range are applied here. **b:** Photographs of the three photodetectors from Supplementary Fig. 8a with a structured background are arranged vertically. Each device consists of four photo-active areas (dark squares) arising from the intersection between vertical and horizontal electrodes. The layer thickness $t_{\mathrm{Ag}}^{\mathrm{bot}}$ of the four vertical electrodes, e.g. the silver bottom electrodes, is simultaneously varied among the three devices. When decreasing $t_{\mathrm{Ag}}^{\mathrm{bot}}$ from 100nm to 15nm, semi-transparency for visible wavelengths is realized. This feature, however, is achieved at the cost of EQE enhancement, due to a reduced number of photon circulations within the microcavity, as shown in Supplementary Fig. 8a. In principle, better visible transparency can be obtained by combining appropriate visibly transparent photo-active blend constituents with dielectric mirrors.

Appearance	Main text							Supplementary information				
Function	Photodetector						Ref.	Photodetector				
Resonance / nm	875	910	950	995	1035	1085	–	910	935	960	$\gtrsim$1100	
Device label	●	■	◆	◀	▶	◓	×	◖	◗	♣	♠	
Active area / mm^2	6.4	6.4	6.4	6.4	6.4	6.4	6.4	6.4	6.4	6.4	0.25	
**Material(s)**	**Layer thickness / nm**							**Layer thickness / nm**				**Function**
Glass	10^6	10^6	10^6	–	–	10^6	10^6	10^6	10^6	10^6	10^6	Substrate
pPEN	–	–	–	$\sim10^5$	$\sim10^5$	–	–	–	–	–	–	
AlO_x	–	–	–	20	20	–	–	–	–	–	–	
MoO_3	–	–	–	3	3	3	–	3	3	3	–	Electrode
Au	–	–	–	1	1	1	–	1	1	1	–	
Ag	100	100	100	100	100	100	100	100	25	15	100	
n-Bis-HFl-NTCDI ⊘	65	71	81	–	–	–	36	–	–	78	–	ETL
BPhen:Cs (1:1)	–	–	–	79	88	105	–	70	70	–	105	
C_{60}	5	5	5	5	5	5	5	5	5	5	5	
ZnPc:C_{60} (1:1)	50	50	50	50	50	50	50	50	50	50	50	D:A blend
ZnPc	5	5	5	5	5	5	5	5	5	5	5	HTL
p-BF-DPB ⊕	63	70	79	75	80	91	31	–	–	–	91	
p-BF-DPB ⊚	–	–	–	–	–	–	–	70	70	70	–	
MoO_3	3	3	3	3	3	3	3	3	3	3	3	Electrode
Au	1	1	1	1	1	1	1	1	1	1	1	
Ag ⊛	18	18	18	25	25	25	9	30	30	30	25	
MoO_3	10	10	10	10	10	10	10	10	10	10	10	
Alq_3	50	50	50	50	50	50	50	50	50	50	50	
ZnPc	200	200	200	200	200	200	–	–	–	–	–	Filter
P4-Ph4-DIP	200	200	200	200	200	200	–	–	–	–	–	
C_{60}	–	–	–	100	100	100	–	–	–	–	–	
Glass	10^6	10^6	10^6	–	–	10^6	10^6	10^6	10^6	10^6	10^6	Top cover
Barrier glue	–	–	–	$\sim10^4$	$\sim10^4$	–	–	–	–	–	–	
AlO_x	–	–	–	20	20	–	–	–	–	–	–	
pPEN	–	–	–	$\sim10^5$	$\sim10^5$	–	–	–	–	–	–	

Supplementary Table 1 | Overview of layer sequence of all investigated ZnPc:C_{60} devices. The layers are deposited reading downwards. Layer thicknesses are given in nm. The ETL matrix Bis-HFl-NTCDI is *n*-doped with 7 wt% $W_2(hpp)_4$ (⊘) and the HTL matrix BF-DPB *p*-doped with 10wt% NDP9 (⊕) or F_6-TCNNQ (⊚), respectively. Furthermore, the thin silver electrode on top is reinforced outside the photo-active area for better electrical contacting by an additional silver layer of 75nm thickness (⊛). 'Ref.' stands for the reference device with thin transport layers and a highly transparent top electrode for minimal interference. The device labeling is used consistently throughout this work.

Function	Photodetector								Ref.	
Resonance / nm	810	950	1060	1160	1280	1360	1480	1550	–	
Variation type	▽	▽	▽	▽	▽	▽	△	△	⬡	
**Material(s)**	**Layer thickness t / nm**								**t / nm**	**Function**
Glass	10^6	10^6	10^6	10^6	10^6	10^6	10^6	10^6	10^6	Substrate
ITO	–	–	–	–	–	–	–	–	90	Electrode
Ag	100	100	100	100	100	100	100	100	–	
BPhen:Cs (1:1)	55	74	91	106	125	140	125	140	20	ETL
C_{60}	5	5	5	5	5	5	5	5	5	
TPDP:C_{60} (1:19)	50	50	50	50	50	50	100	100	50	D:A blend
p-BF-DPB ◎	55	74	92	106	126	140	126	140	–	HTL
p-m-MTDATA ◎	–	–	–	–	–	–	–	–	30	
F_6-TCNNQ	–	–	–	–	–	–	–	–	1	
MoO_3	3	3	3	3	3	3	3	3	–	Electrode
Au	1	1	1	1	1	1	1	1	–	
Ag ⊛	25	25	25	25	25	25	25	25	–	
MoO_3	10	10	10	10	10	10	10	10	–	
Alq_3	50	50	50	50	50	50	50	50	–	
Al	–	–	–	–	–	–	–	–	100	
Glass	10^6	10^6	10^6	10^6	10^6	10^6	10^6	10^6	10^6	Top cover

Supplementary Table 2 | Overview of layer sequence of all investigated TPDP:C_{60} devices. The layers are deposited reading downwards. Layer thicknesses are given in nm. For the cavity enhanced devices, the resonance wavelength is varied from 810nm to 1550nm by thickness variations of both transport layers (▽) and for selected devices as well by a thickness variation of the TPDP:C_{60} blend (△). The matrix of the hole transport layer BF-DPB or m-MTDATA is *p*-doped with 10wt% of F_6-TCNNQ (◎) where the latter matrix material stands for 4,4',4''-tris(3-methylphenylphenylamino)-triphenylamine. 'Ref.' stands for the reference device with thin transport layers and a highly transparent top electrode for minimal interference. Furthermore, the thin silver electrode on top is reinforced outside the photo-active area for better electrical contacting by an additional silver layer of 75nm thickness (⊛). All samples exhibit a photo-active area of 6.4mm^2. The device labeling is used consistently throughout this work.

Supplementary Discussion

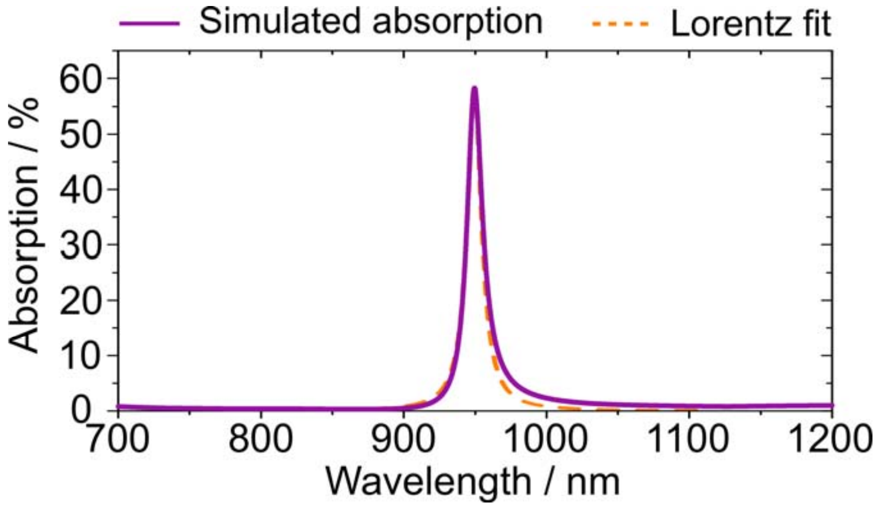

Supplementary Figure 9 | Spectral detection limit for silver cavities. Simulated absorption spectrum (solid line) of a silver microcavity in absence of an absorber with a resonance wavelength of λ_{res}=950nm. The silver mirrors of 25nm and 100nm layer thickness incorporate 230nm of an organic high bandgap material with index of refraction of 1.7 as fulfilled for many transport matrix materials. The dashed line reads a Lorentz fit of $\Delta\lambda$ =13nm spectral width and corresponds to a quality factor of $Q = \lambda_{res}/\Delta\lambda = 73$.

The weak absorption of CT states is crucial both to the EQE FWHM and height. In absence of any substantial absorption between the two silver mirrors, at a resonance wavelength in the NIR, spectral widths below 20nm can be achieved, as shown by the simulation in Supplementary Fig. 9. Hereby, the narrowband absorption is achieved due a quality factor Q of about 70 for the simulated resonator. In order to keep the high quality factor, resulting both in high EQE amplifications and narrow linewidths, the absorption coefficient of the active layer needs to be comparably small. Otherwise, the cavity resonance breaks down, leading to a flat and unamplified signal. This mechanism makes the weak absorption, provided for example by CT states, so attractive for use in a tunable microcavity detector. In contrast to other weakly absorbing states, such as absorption tails, CT bands provide the following advantages:

1. CT states can exhibit extremely broad absorption bands due to the disordered nature of non-crystalline organics: Compare for instance the tunability range over more than 700nm for the TPDP:C_{60} system in Figure 5a of the main paper.
2. The CT absorption onset, determined the frontier energy levels of both D and A, can be tuned continuously over several *eV* via appropriate blending of the large variety of small molecules and/or polymers.
3. Upon an appropriate choice materials resulting in a further reduced CT energy, the current detection limit for organics can be pushed further into the infrared.
4. As the CT energetics is determined neither by the donor LUMO nor acceptor HOMO level, the optical gaps of the blend constituents remain as degree of freedom. When exploiting D:A blends of high gap materials, we expect the detectors to become intrinsically visible blind and, therefore, the external filter layers as an intermediate step.

Supplementary Note 1

Details on electrodes

The circumstance that a single metal type can be applied simultaneously for both electrodes without extraction losses traces back to the usage of doped transport layers. Hereby, an appropriate doping causes an alignment of the respective frontier orbital energy with the work function of the metal.[1] This mechanism, finally, leads to the build-up of an internal electrical field, guiding the charges to their respective electrodes.

As shown in Supplementary Table 1 and 2, the top electrode comprises a multilayer sequence: The silver layer is grown onto a diffusion barrier of 3nm MoO_3 to ensure an efficient charge extraction and to prevent the diffusion of metal atoms into the organic films below. A seed layer of 1nm Au is used to overcome the gradient in surface energy between MoO_3 and Ag and, thus, to obtain a smooth Ag thin film instead of islands.[2] For enhanced light incoupling into the microcavity and protection of the underlying layers, 10nm of MoO_3 and 50nm of Alq_3 are grown as capping layers.[3] In addition, both MoO_3 layers embedding the top silver film are expected to passivate it against most degradation paths.[3]

Details on transport layers

5nm of the respective intrinsic absorber may be inserted between the doped transport layer and the absorber blend. This procedure prevents diffusion of the dopants into the latter layer, suppressing potential exciton quenching.

Supplementary Methods

External quantum efficiency

To examine devices with blended photo-active layers, a xenon or quartz halogen lamp is combined with a monochromator and a chopper. The resulting spectrally tunable, pulsed excitation light illuminates the samples via the transparent electrode. A lock-in amplifier resolves the pre-amplified photocurrent. A calibrated silicon reference diode and an optional indium gallium arsenide photodiode monitor the excitation intensity. The spectra in the absorption tail region of neat ZnPc and C_{60} reference samples are investigated via Fourier transform photocurrent spectroscopy (FTPS) as described in Reference [4] in continuous scan mode.

Specific detectivity

Current-voltage characteristics are recorded with a source measurement unit (2400 SourceMeter, Keithley Instruments, USA). If required, an inorganic light emitting diode with a peak emission wavelength of 905nm (H2W5-905, Roithner Lasertechnik, Germany) illuminates the sample with an intensity of $2mWcm^{-2}$. In general, the noise spectral density i_{noise} constitutes as sum of shot, thermal, 1/f and generation-recombination noise [5]. With the highest detectivity at zero bias, we estimate the thermal noise to dominate in absence of an external voltage [6-10]: $i_{noise} = (4k_B T/R_{sh})^{1/2}$ with k_B as Boltzmann constant, T as temperature, and R_{sh} as differential shunt resistance at short-circuit. Based on analyzing the dark current-voltage characteristics, which are exemplarily shown in Supplementary Fig. 8a, we obtain $10^{-13}AHz^{-1/2}$ for all ZnPc:C_{60} devices at room temperature, whereas the optimization strategies outlined in the main text are expected to improve the noise behavior. The specific detectivity is determined as $D^*=(e\lambda A^{1/2}\ EQE) / (hc\ i_{noise})$ with e as elementary charge, λ as excitation wavelength, A as photo-active area, h as Planck constant, and c as speed of light.[6,7]

Absorption

The absorption *A* under quasi-normal light incidence is determined via a measurement of the directly reflected light beam *R* (SolidSpec 3700, Shimadzu, Japan) according to *A=1-R*. Hereby, the transmission *T* through the silver film of 100nm thickness can be neglected (*T=0*). For the angular dependence of the absorption, a goniometer setup as described in Reference [11] is used in reflection mode.

Optical simulation

For predicting the internal optical field distribution and optimizing the absorption in the photo-active blend for a particular resonance wavelength, we apply the transfer-matrix algorithm as described in Reference [12]. The required optical constants of all contributing layers are obtained upon modelling the experimental, direct reflection and transmission (UV-3100 Spectrometer, Shimadzu, Japan) of thin films with varying layer thickness.

Linear dynamic range

An inorganic light emitting diode peaking at 905nm (H2W5-905, Roithner Lasertechnik, Germany) hits the device through its thinner, semi-transparent silver electrode. A sequence of neutral density filters controls the excitation intensity. For intensities up to 3mWcm^{-2}, the light is chopped at 230Hz and a lock-in amplifier (Model 7265, Signal Recovery, USA) resolves the pre-amplified (10^{-5}A/V, Model 5182, Signal Recovery, USA) photo-current. For intensities above 3mWcm^{-2}, the photocurrent is recorded with an SMU (2400 SourceMeter, Keithley Instruments, USA). A calibrated silicon reference diode (S1337, Hamamatsu, Japan) monitors the respective excitation intensities.

Transient photo current

A Nd-YAG laser (PL2210, Ekspla, Lithuania) generates 25ps pulses at a wavelength of 1064nm at a repetition rate of 10Hz. An oscilloscope (DPO7354C, Tektronix, USA) records the temporal photocurrent decay of the sample which is directly connected to an internal 50Ω impedance leading to an overall circuit resistance of 100Ω.

Supplementary References

[1] Blochwitz, J. *et al*. Interface electronic structure of organic semiconductors with controlled doping levels. *Organic Electronics* **2**, 97–104 (2001).

[2] Schubert, S., Meiss, J., Müller-Meskamp, L. & Leo, K. Improvement of transparent metal top electrodes for organic solar cells by introducing a high surface energy seed layer. *Advanced Energy Materials* **3**, 438–443 (2013).

[3] Schubert, S., Hermenau, M., Meiss, J., Müller-Meskamp, L. & Leo, K. Oxide sandwiched metal thin-film electrodes for long-term stable organic solar cells. *Advanced Functional Materials* **22**, 4993–4999 (2012).

[4] Vandewal, K. *et al*. Fourier-Transform Photocurrent Spectroscopy for a fast and highly sensitive spectral characterization of organic and hybrid solar cells. *Thin Solid Films* **516**, 7135–7138 (2008).

[5] Jansen-van Vuuren, R. D., Armin, A., Pandey, A. K., Burn, P. L. & Meredith, P. Organic Photodiodes: The Future of Full Color Detection and Image Sensing. *Advanced Materials* **28**, 4766–4802 (2016).

[6] Zimmerman, J. D. et al. Porphyrin-Tape/C_{60} Organic Photodetectors with 6.5% External Quantum Efficiency in the Near Infrared. *Advanced Materials* **22**, 2780–2783 (2010).

[7] Zimmerman, J. D. *et al*. Use of additives in porphyrin-tape/C_{60} near-infrared photodetectors. *Organic Electronics: Physics, Materials, Applications* **12**, 869–873 (2011).

[8] Su, Z. *et al*. High-performance organic small-molecule panchromatic photodetectors. ACS *Applied Materials and Interfaces* **7**, 2529–2534 (2015).

[9] Wang, X. *et al*. Efficient organic near-infrared photodetectors based on lead phthalocyanine/C_{60} heterojunction. *Organic Electronics: physics, materials, applications* **15**, 2367–2371 (2014).

[10] Zhu, H. *et al*. Metal-oxide-semiconductor-structured MgZnO ultraviolet photodetector with high internal gain. *Journal of Physical Chemistry C* **114**, 7169–7172 (2010).

[11] Fiehler, V. *et al*. Plasmonic Nanorod Antenna Array: Analysis in Reflection and Transmission. *The Journal of Physical Chemistry C* **120**, 12178–12186 (2016).

[12] Pettersson, L. A. A., Roman, L. S. & Inganäs, O. Modeling photocurrent action spectra of photovoltaic devices based on organic thin films. *Journal of Applied Physics* **86**, 487 (1999).
